# Microwave-Assisted Synthesis, Cytotoxic Profile, and Cellular Mechanism Analysis of Carveoylphenol Derivatives

**DOI:** 10.3390/ijms27198749

**Published:** 2026-09-30

**Authors:** Evelyn Muñoz, Constanza Reyes, Joan Villena, Rut Vergara, Guillermo Bravo, Alejandro Madrid

**Affiliations:** 1 Facultad de Ciencias Químicas, Departamento de Química Orgánica, Universidad de Concepción, Concepción 4030000, Chile; emunoznu@udec.cl; 2 Laboratorio de Productos Naturales y Síntesis Orgánica (LPNSO), Facultad de Ciencias Naturales y Exactas, Universidad de Playa Ancha, Avda. Leopoldo Carvallo 270, Playa Ancha, Valparaíso 2340000, Chile; reyesveraconstanza@gmail.com; 3 Center of Interdisciplinary Biomedical and Engineering Research for Health (MEDING), Escuela de Medicina, Universidad de Valparaíso, Angamos 655, Reñaca, Viña del Mar 2340000, Chile; juan.villena@uv.cl (J.V.); rut.vergara@uv.cl (R.V.); 4 Departamento de Química y Medio Ambiente, Universidad Técnica Federico Santa Maria, Avenida Federico Santa Maria 6090, Viña del Mar 2520000, Chile; guillermo.bravo@usm.cl

**Keywords:** carveol, terpenophenols, apoptosis, reactive oxygen species, mitochondrial membrane potential, molecular docking

## Abstract

A series of carveoylphenol derivatives (**1**–**12**) was synthesized using a microwave-assisted approach and evaluated through an integrated in vitro and in silico strategy. Cytotoxicity was assessed against PC-3, MCF-7, and HT-29 human cancer lines, using non-tumorigenic CoN CCD841 cells as a reference. Derivatives **1**, **2**, **5**, **6**, **7**, **8**, and **11** exhibited activity (IC_50_ < 100 µM) against at least one cancer line, with **2** showing the highest potency (IC_50_ = 23.2 µM). Flow cytometry of compounds **1**, **2**, and **5** revealed structure-dependent cellular responses, where **2** at 40 µM induced maximum intracellular ROS generation (39.6%), **1** reduced mitochondrial membrane integrity to 42.1% intact cells, and **5** produced peak caspase activation (34.5%). Molecular docking against caspase-3 yielded predicted binding energies for all three molecules, with **5** scoring −6.4 kcal/mol. SwissADME analysis indicated comparable physicochemical profiles, high predicted gastrointestinal absorption, and potential blood–brain barrier permeability for these three derivatives. Overall, carveoylphenols, particularly compound **2**, represent promising scaffolds for further structural optimization and mechanistic investigation.

## 1. Introduction

Cancer remains a primary cause of global morbidity and mortality [1,2]. Although advances in chemotherapy, radiotherapy, immunotherapy, and targeted treatments have improved clinical outcomes, therapeutic efficacy is frequently limited by off-target toxicity [3], non-specific cytotoxicity, and multidrug resistance [4,5,6]. Driven by the molecular heterogeneity of solid tumors, these limitations highlight the need for novel chemical entities with distinct biological mechanisms [7].

Natural products and their synthetic derivatives represent a major source for oncology drug discovery [8], as exemplified by paclitaxel, vincristine, and camptothecin analogues [2,9]. Plant secondary metabolites with phenolic scaffolds are particularly relevant due to their structural versatility and capacity to modulate key oncogenic pathways, including redox homeostasis, cell proliferation, and apoptotic cascades [10,11,12]. Consequently, phenolic structures serve as effective building blocks for hybridization with other bioactive pharmacophores.

Carveol, a monocyclic monoterpene alcohol found in essential oils, displays antioxidant, antimicrobial, and antiproliferative activities [13,14,15,16]. This monoterpenoid induces *S*-phase cell-cycle arrest in cancer models [17], and its functionalization with 4-(2-methoxyphenyl)piperazine moieties increases cytotoxicity against LNCaP prostate cancer cells [18]. Recently, carveol has served as a monoterpenoid building block for the synthesis of terpenophenols [19]. For instance, coupling carveol with phenolic substrates yielded carveoylphenols with antifungal activity against *Candida* species [19]. However, the cytotoxic profile, structure–activity relationships, and underlying cellular mechanisms of carveoylphenols against human cancer cell lines have not been established.

Microwave-assisted organic synthesis (MAOS) is an established approach in medicinal chemistry, facilitating rapid library generation with improved reaction kinetics, yields, and energy efficiency relative to conventional heating [20,21,22,23]. Combined with heterogeneous catalysis, MAOS offers a sustainable protocol for functionalizing natural product derivatives [24,25]. Applying a microwave-assisted catalytic strategy thus provides an efficient route for synthesizing and systematically evaluating carveoylphenol derivatives.

Small-molecule cytotoxicity frequently involves the disruption of organelle function and redox homeostasis [26]. Intracellular reactive oxygen species (ROS) overproduction can induce mitochondrial membrane depolarization, triggering downstream apoptotic signaling and caspase activation [27,28,29]. Quantifying ROS generation, mitochondrial membrane integrity, and caspase execution provides mechanistic insight into compound-induced cell death.

In this study, a series of carveoylphenol derivatives (**1**–**12**) were synthesized using a microwave-assisted approach. The cytotoxic profile of these derivatives was evaluated in vitro against PC-3, MCF-7, and HT-29 human cancer lines, alongside non-tumorigenic CoN CCD841 reference cells. Cellular mechanism analysis was conducted via flow cytometry to assess intracellular ROS generation, mitochondrial membrane integrity, and caspase activation. Finally, molecular docking against caspase-3 and SwissADME predictions were performed to explore binding interactions and pharmacokinetic profiles.

## 2. Results and Discussion

### 2.1. Synthesis of Carveoylphenols

**Scheme 1:**
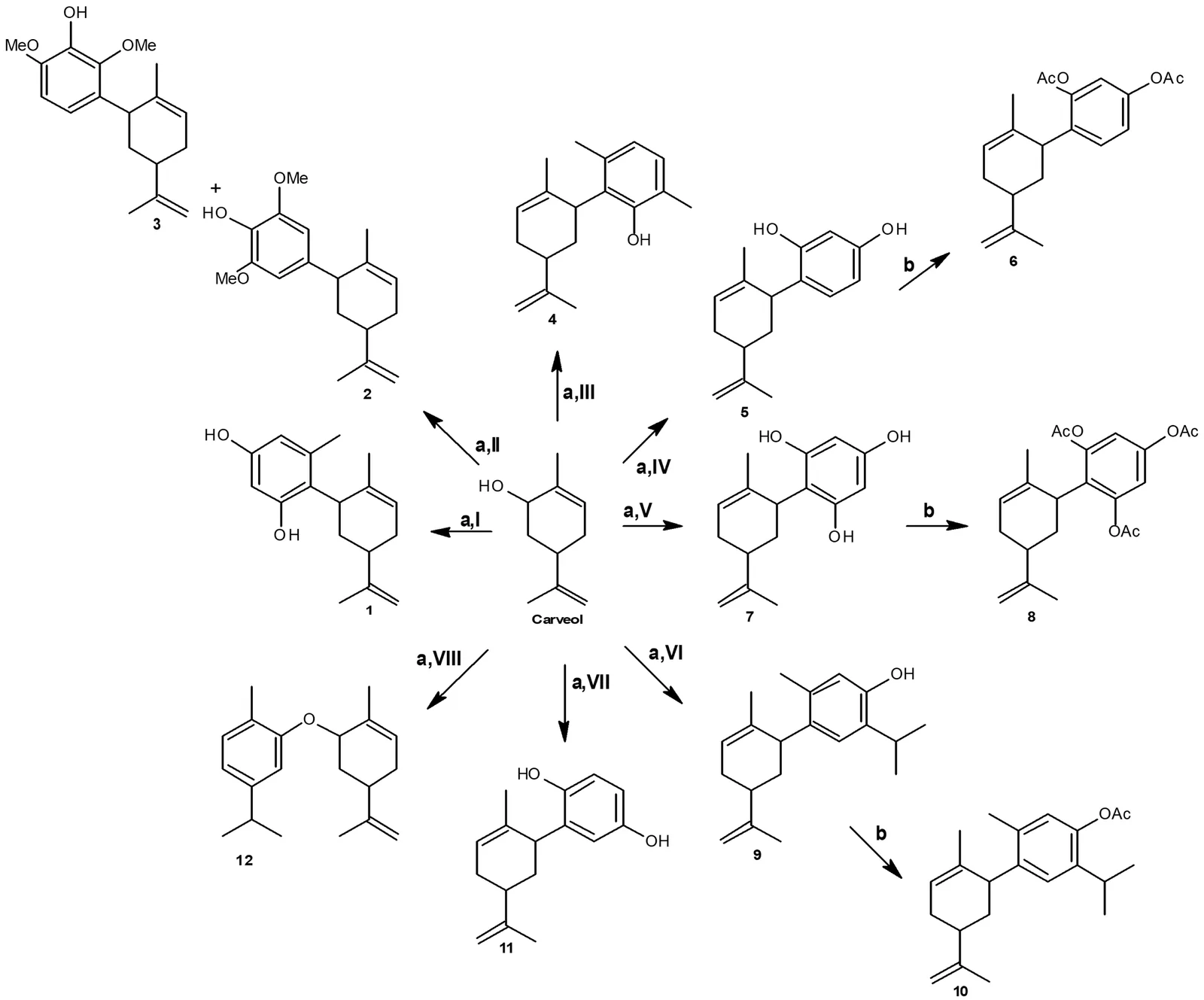
Reagents and conditions: (**a**) carveol, phenolic derivatives **I**–**VIII** (2:1 molar ratio), AgNO_3_/SiO_2_ (20% *w*/*w*), MW (25 W, 55 °C), 40 min; (**b**) for crude mixtures containing **5**, **7**, or **9**: Ac_2_O (1.0–3.0 equiv.), DMAP (cat.), dry CH_2_Cl_2_, rt, 2 h. Phenolic derivatives: **I** = orcinol, **II** = 2,6-dimethoxyphenol, **III** = 2,5-dimethylphenol, **IV** = resorcinol, **V** = phloroglucinol, **VI** = thymol, **VII** = hydroquinone, **VIII** = carvacrol. Yields: **1** (44.1%), **2** (51.3%), **3** (27.0%), **4** (41.2%), **5** (61.0%), **6** (94.0%), **7** (49.1%), **8** (92.0%), **9** (50.7%), **10** (93.2%), **11** (54.0%), 12 (26.6%).

The direct alkylation of phenols with monoterpenoid allylic alcohols provides efficient access to bioactive terpenophenols [19]. However, conventional methodologies using liquid Lewis acids (e.g., BF_3_ OEt_2_ or TiCl_4_) often require prolonged reaction times or high temperatures, frequently yielding complex mixtures due to side reactions such as retro-Friedel–Crafts rearrangements or cyclizations into pyran derivatives [19,30]. To overcome these drawbacks through a greener and faster approach, a microwave-assisted heterogeneous catalytic manifold was developed, as outlined in Scheme 1.

The coupling of carveol with phenolic derivatives **I**–**VIII** (2:1 molar ratio) under the proposed catalytic system provided the corresponding carveoylphenol adducts **1**–**12** with an overall yield improvement of approximately 7%. Building upon the AgNO_3_/SiO_2_ catalytic methodology previously reported [31], implementing an automated, single-mode microwave system under fixed power conditions (25 W, 55 °C, 40 min) drastically accelerated the alkylation process compared to discontinuous heating in domestic microwave ovens. Reducing the reaction time to just 40 min compared to previous Lewis acid protocols using TiCl_4_/Al_2_O_3_ or classical conductive heating that typically require 24 h or up to 48 h [19,30,31,32]. Furthermore, the heterogeneous support restricted secondary intramolecular rearrangements, preventing the formation of undesired pyran-fused or cyclized byproducts [19,33]. This microwave-assisted strategy facilitates access to functionalized carveoylphenolic frameworks, a class of compounds recognized for their relevant biological properties, including antifungal and antimicrobial potential [19,32].

The reaction exhibited marked regioselectivity, taking place selectively at the allylic C-1′ position of the carveol moiety and connecting preferentially to the position *ortho* or *para* relative to the phenolic hydroxyl group [19,32]. For electron-rich substrates such as orcinol (**I**), resorcinol (**IV**), phloroglucinol (**V**), and hydroquinone (**VII**), electrophilic attack proceeded smoothly to yield the corresponding *C*-alkylated derivatives [19,32]. In addition, the acetylated adduct derived from phloroglucinol (derivative **8**) and the *O*-alkylated derivative obtained from carvacrol (derivative **12**) represent novel chemical entities described here for the first time, whereas derivatives 1–7 and 9–11 correspond to previously reported analogs [19,33,34].

A remarkable divergence in reactivity was observed between the constitutional isomers thymol (**VI**) and carvacrol (**VIII**): while thymol cleanly underwent *C*-alkylation, carvacrol selectively afforded the novel *O*-alkylated carveoyl ether [30]. This behavior is rationalized by the severe steric congestion surrounding the aromatic ring in carvacrol due to the adjacent methyl and isopropyl substituents, which significantly elevates the activation barrier for electrophilic aromatic substitution (*C*-alkylation), rendering nucleophilic attack through the phenolic oxygen (*O*-alkylation) the kinetically favored pathway [30]. In the case of 2,6-dimethoxyphenol (**II**), both the major para-alkylation product and minor ortho-substituted isomers were obtained [30,32], confirming the strong activating capability of the AgNO_3_/SiO_2_ system even on sterically hindered or electronically deactivated cores.

Direct isolation and purification of the crude reaction mixtures containing polyhydroxylated or highly reactive cores, specifically those yielding derivatives **5**, **7**, and **9**, proved challenging due to high polarity, partial oxidation, and heavy steric shielding around the polyphenolic core [30]. To overcome these constraints and significantly increase overall isolation yields, these specific crude mixtures were subjected to direct derivatization via acetylation using acetic anhydride [30]. This transformation successfully converted the reactive phenolic groups into their corresponding acetate esters, facilitating clean chromatographic separation, structural stabilization, and unambiguous spectroscopic characterization of derivatives **5**, **7**, and **9** [30]. In contrast, attempting acetylation on the complex reaction mixtures derived from the other phenols proved unsuccessful or ineffective for product recovery, leaving this derivatization strategy effective and applicable exclusively for the optimization and isolation of derivatives **5**, **7**, and **9** [19,30,31,32,33]. Additionally, the percentage of residual carveol varies according to the nucleophilicity of the specific phenol used, indicating that isolated yield variations reflect product stability and recovery constraints during chromatography rather than incomplete conversion. Furthermore, the catalytic system strictly requires allylic alcohols capable of generating silver-stabilized carbocations, leaving aliphatic alcohols and non-allylic nucleophiles entirely unreactive. Finally, this protocol extends smoothly to other structurally diverse allylic alcohols, as previously demonstrated [30,31,32,33].

### 2.2. Cytotoxic Activity of Carveoylphenols

The cytotoxic activity of the synthesized carveoylphenols was evaluated against the human cancer cell lines PC-3, MCF-7, and HT-29, using the non-tumorigenic colon cell line CCD841 as a reference model. The half-maximal inhibitory concentration (IC_50_) values obtained for compounds **1**–**12**, isocordoin, and the reference controls are presented in Table 1.

The carveoylphenols exhibited cytotoxicity profiles that varied according to both chemical structure and cell line. Compounds **1**, **2**, **5**, **6**, **7**, **8**, and **11** showed cytotoxic activity (IC_50_ < 100 µM) against at least one tumor cell line, whereas compounds **4**, **10**, and **12** displayed IC_50_ values >100 µM across all tested cell lines. Compound **3** showed limited activity, with an IC_50_ value of 95.2 ± 4.7 µM against CCD841 and values >100 µM against the tumor cell lines. In contrast, compound **9** exhibited low activity against PC-3 and MCF-7 (IC_50_ > 100 µM), but moderate activity against HT-29 (85.1 ± 17.9 µM).

Among the synthesized derivatives, compound **2** exhibited the highest cytotoxic activity, with IC_50_ values of 26.5 ± 6.2, 30.9 ± 3.3, and 23.2 ± 5.0 µM against PC-3, MCF-7, and HT-29, respectively, followed by compound **6**, with IC_50_ values ranging from 34.5 ± 1.7 to 47.2 ± 5.2 µM. Compounds **1**, **5**, **7**, **8**, and **11** showed intermediate activity, with IC_50_ values generally ranging from 46.4 to 79.0 µM, although compound **8** exhibited an IC_50_ >100 µM against MCF-7.

In comparison, isocordoin exhibited greater cytotoxic potency against all three tumor cell lines, with IC_50_ values of 15.2 ± 0.6, 21.1 ± 0.2, and 27.2 ± 0.3 µM against PC-3, MCF-7, and HT-29, respectively. However, its cytotoxicity toward CoN was lower (78.7 ± 0.1 µM). The commercial controls daunorubicin and 5-fluorouracil also exhibited cytotoxic activity, although their potency differed among the cell lines.

The observed variability suggests that the nature and position of the phenolic substituent may modulate the antiproliferative activity of the carveoylphenols. Compounds **2** and **6** exhibited the highest activity among the synthesized derivatives. This structure-dependent behavior is consistent with previous studies indicating that functionalization of monoterpene scaffolds can substantially influence their antitumor activity, depending on the functional groups introduced and the cellular model employed [35]. Likewise, the differential activity reported for phenolic and terpenoid compounds across tumor cell lines supports the combined influence of chemical structure and cellular context on the cytotoxic response [36].

The selectivity of the derivatives toward tumor cells relative to the non-tumorigenic CoN cell line was assessed using the selectivity index (SI), calculated as the ratio of the IC_50_ value obtained for CoN to that determined for each tumor cell line. The results are presented in Table 2.

Among the synthetic derivatives, compound **2** exhibited the highest SI values, although these remained below the established selectivity threshold (SI > 2), with values of 1.21, 1.04, and 1.38 against PC-3, MCF-7, and HT-29, respectively. Compounds **5** and **6** also showed SI values >1 against some tumor cell lines, particularly HT-29 (1.27 and 1.09, respectively). In contrast, compounds **6**, **7**, **8**, and **11** exhibited SI values <1 against PC-3 and MCF-7, indicating greater relative cytotoxicity toward non-tumorigenic cells. For derivatives with IC_50_ values >100 µM, SI values were expressed as limits or were not determined.

In comparison, isocordoin displayed marked selectivity, with SI values of 5.18, 3.73, and 2.89 against PC-3, MCF-7, and HT-29, respectively. Thus, although compound **2** was the most potent synthetic derivative, none of the carveoylphenols met the established selectivity criterion, suggesting that their cytotoxic activity was not clearly preferential toward tumor cells over CoN. This contrast with isocordoin highlights the complementary importance of cytotoxic potency and selectivity in the evaluation of new anticancer candidates, particularly natural product-derived compounds [35].

Considering both cytotoxic potency and selectivity profiles, compounds **1, 2,** and **5** were selected for subsequent flow cytometry analyses. These assays were used to investigate potential cellular mechanisms underlying their cytotoxic effects by assessing intracellular ROS generation, mitochondrial membrane integrity, and caspase activation.

### 2.3. Effects of the Compounds on ROS Generation, Mitochondrial Integrity, and Caspase Activation

To investigate the potential mechanisms underlying the cytotoxic activity of compounds **1**, **2**, and **5**, flow cytometry was used to assess reactive oxygen species (ROS) generation, mitochondrial membrane integrity, and caspase activation.

**Figure 1:**
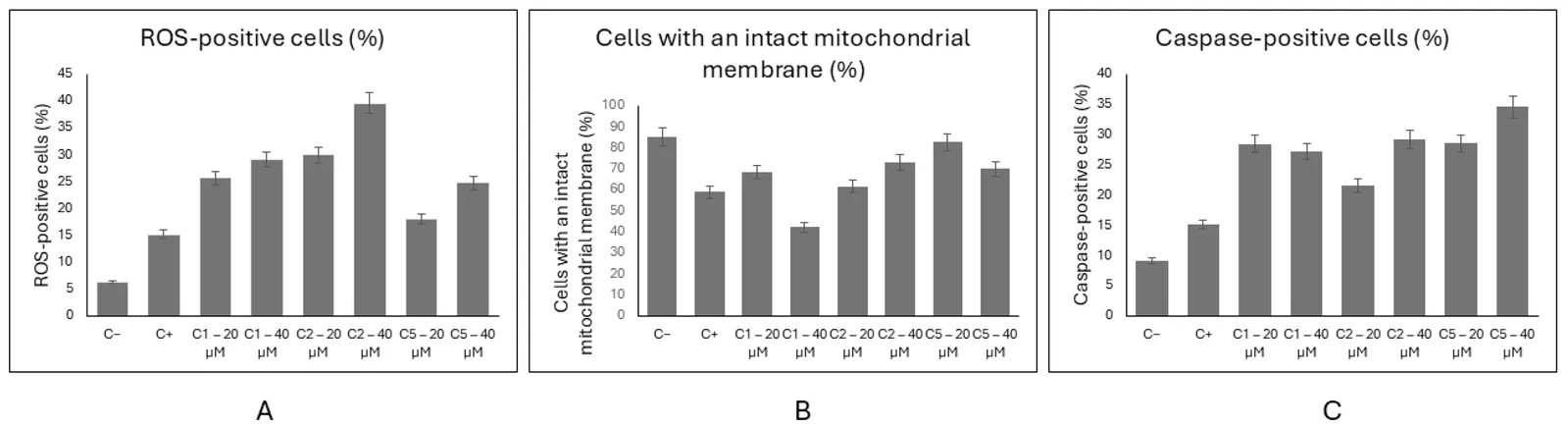
Flow cytometric assessment of ROS generation, mitochondrial membrane integrity, and caspase activation induced by the carveoylphenols. (**A**) Percentage of ROS-positive cells; (**B**) percentage of cells with intact mitochondrial membranes; and (**C**) percentage of caspase-positive cells after treatment with compounds **1**, **2**, and **5** at 20 and 40 µM. Data are expressed as mean ± standard deviation. *p* < 0.001 vs. vehicle control (C+).

As shown in Figure 1, the percentage of ROS-positive cells was 6.3% in the C− control and 15.2% in the C+ control. All three derivatives increased the ROS-positive population relative to C+. Compound **1** yielded 25.6% and 29.1% ROS-positive cells at 20 and 40 µM, respectively, whereas compound **2** increased this population to 29.9% and 39.6%. Compound **5** produced lower values of 18.0% and 24.7% at the same concentrations. Thus, compound **2** at 40 µM induced the highest ROS generation, with a concentration-dependent response. All treatments differed significantly from C+ (*p* < 0.001).

The increase in ROS suggests an alteration of cellular redox homeostasis, particularly in response to compound **2**. This finding is consistent with previous reports describing the ability of monoterpenes and phenolic compounds to modulate cellular oxidative balance and, depending on their structure and concentration, induce a pro-oxidant state associated with cellular damage [37,38]. The pronounced ROS generation induced by compound **2** is also consistent with its greater cytotoxic potency among the derivatives evaluated.

Mitochondrial membrane integrity was also differentially affected by the compounds (Figure 1). The C− control showed 85.1% of cells with an intact mitochondrial membrane, whereas this proportion decreased to 58.8% in C+. Compound **1** produced the most pronounced effect, reducing the proportion of cells with intact mitochondrial membranes from 68.3% at 20 µM to 42.1% at 40 µM. Compounds **2** and **5** yielded 61.6% and 73.1%, and 82.6% and 69.8% intact cells at 20 and 40 µM, respectively. All treatments showed significant differences relative to C+.

Among the derivatives, compound **1** at 40 µM caused the greatest disruption of mitochondrial integrity, whereas compound **5** exhibited the weakest effect, particularly at 20 µM. Compound **2** showed an intermediate response without a clear concentration-dependent pattern. Because mitochondrial dysfunction can contribute to alterations in membrane potential, ROS production, and activation of cell-death pathways [39], these findings suggest that the derivatives differentially affect mitochondrial homeostasis. However, the extent of mitochondrial disruption did not directly correlate with cytotoxic potency, as compound **2** was the most cytotoxic derivative, whereas compound **1** produced the greatest mitochondrial alteration.

Caspase activation was likewise increased following treatment with the compounds. The proportion of caspase-positive cells was 9.1% in C− and 15.1% in C+. Compound **1** yielded 28.4% and 27.2% positive cells at 20 and 40 µM, respectively, while compound **2** produced 21.6% and 29.2%. Compound **5** showed 28.5% and 34.5%, respectively. Thus, compound **5** at 40 µM induced the highest caspase activation (34.5%), followed by compound **2** at 40 µM (29.2%).

The greater caspase activation induced by compound **5** suggests the involvement of cell-death pathways associated with caspase activation. Caspases play a central role in the execution of apoptosis and have been implicated in the antitumor effects of numerous natural products and their derivatives [40]. Nevertheless, caspase activation did not directly reflect cytotoxic potency, since compound **2** exhibited the lowest IC_50_ values, whereas compound **5** produced the highest proportion of caspase-positive cells.

Overall, flow-cytometric analysis revealed that the carveoylphenols modulated distinct cellular events associated with cytotoxicity in a structure-dependent manner. Compound **2**, the most cytotoxic derivative, produced the greatest increase in ROS (39.6% at 40 µM), compound **1** caused the strongest disruption of mitochondrial integrity (42.1% intact cells at 40 µM), and compound **5** induced the highest caspase activation (34.5% at 40 µM). The lack of a direct correlation between individual cellular parameters and cytotoxic potency suggests that the observed activity may involve multiple, partially convergent mechanisms, including oxidative stress, mitochondrial dysfunction, and caspase-mediated cell death [39,40].

### 2.4. Molecular Docking Study

**Figure 2:**
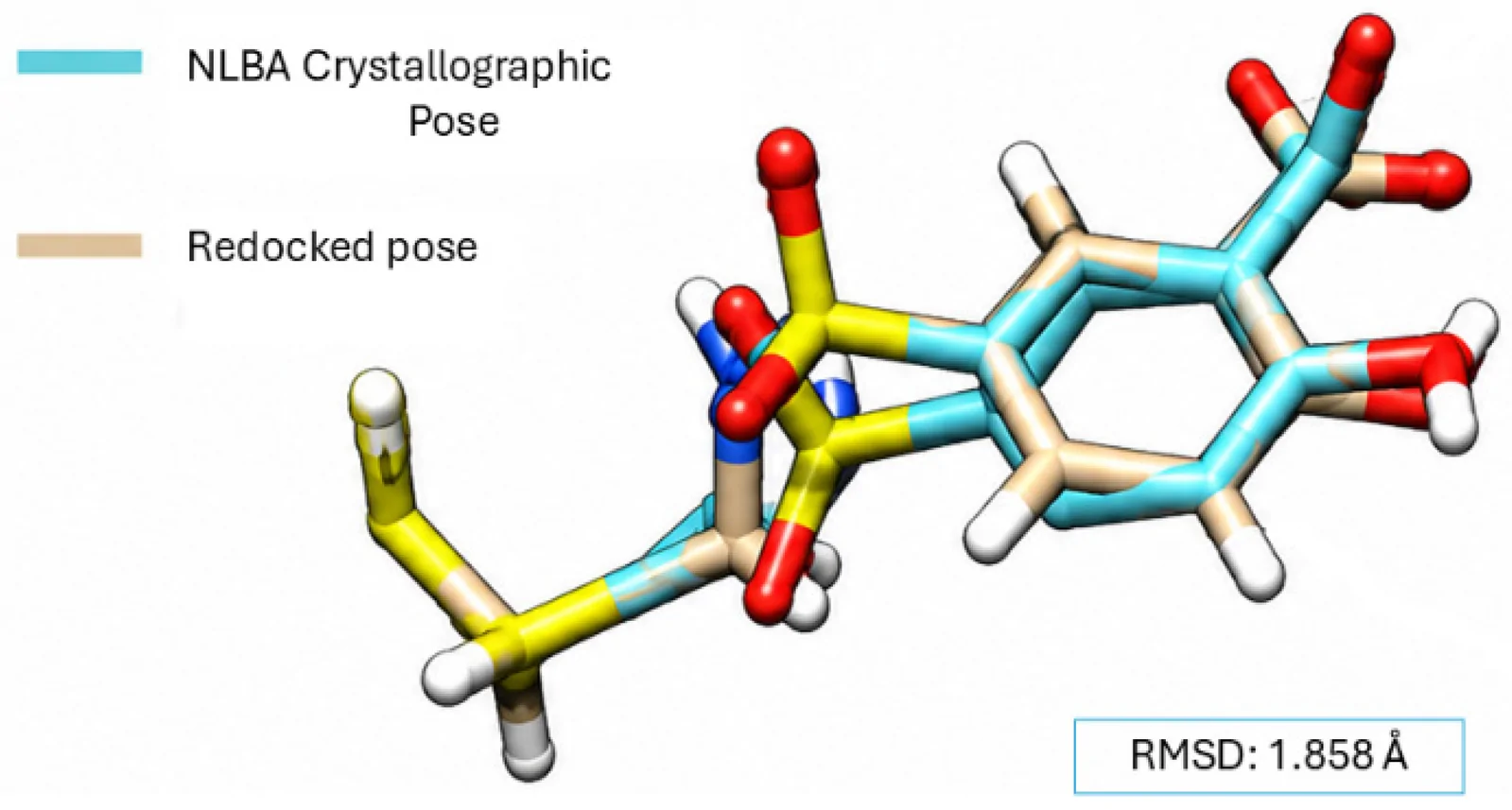
Redocking of the crystallographic ligand into the active site of caspase-3.

**Figure 3:**
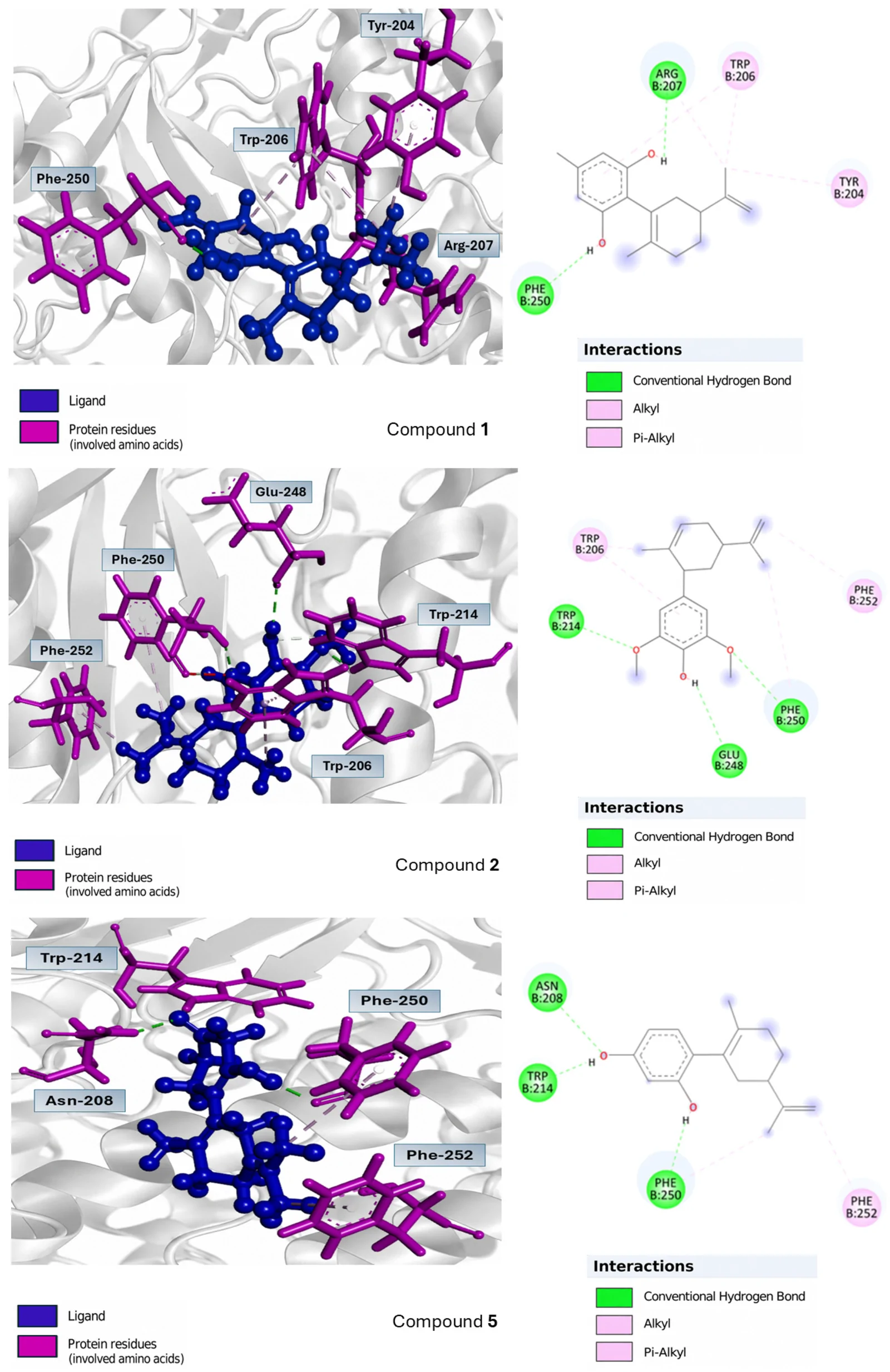
Three-dimensional and two-dimensional interactions of compounds **1**, **2**, and **5** with the caspase-3 binding site.

**Figure 4:**
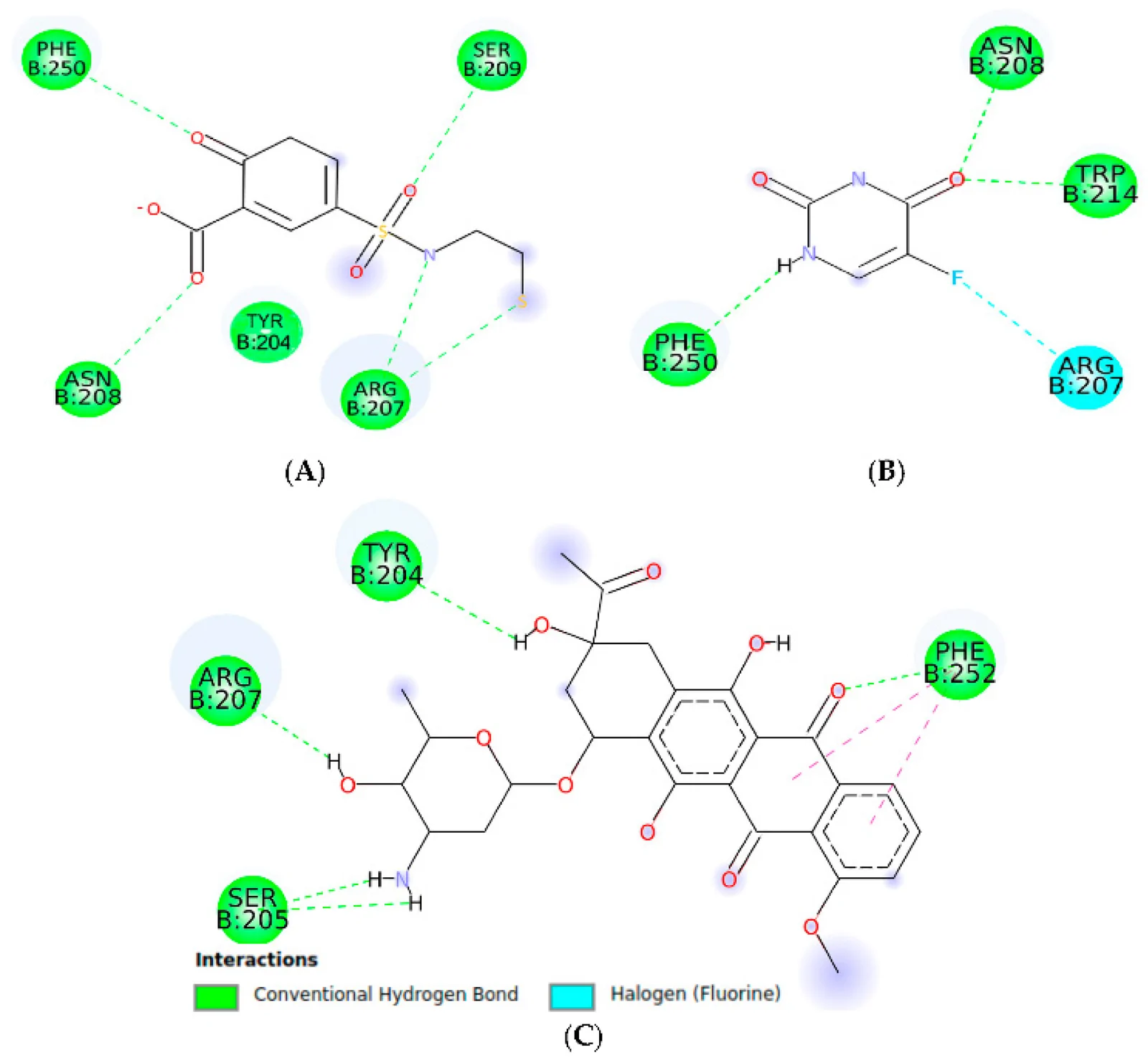
Two-dimensional interactions of 2-hydroxy-5-(2-mercaptoethylsulfamoyl)benzoic acid (**A**), 5-FU (**B**), and daunorubicin (**C**) with the caspase-3 binding site.

To explore the potential molecular interactions associated with the experimentally observed caspase activation, a molecular docking study of compounds **1**, **2**, and **5** against caspase-3 was performed using the crystallographic structure 1NME. The results are summarized in Table 3 and Figure 2, Figure 3 and Figure 4.

The three compounds exhibited favorable predicted binding energies, with values of −6.0, −6.1, and −6.4 kcal/mol for compounds **1**, **2**, and **5**, respectively. Among the derivatives evaluated, compound **5** showed the most favorable docking score (−6.4 kcal/mol), followed by compounds **2** (−6.1 kcal/mol) and **1** (−6.0 kcal/mol). For comparison, isocordoin exhibited a binding energy of −6.1 kcal/mol, whereas NLBA, the ligand associated with the crystallographic structure, showed a value of −5.5 kcal/mol.

The interaction analysis showed that all three compounds were located within a region of the caspase-3 binding site characterized predominantly by aromatic and charged residues. Compound **1** exhibited interactions with Phe250, Trp206, and Arg207, including two putative hydrogen bonds with Phe250 and Arg207, at distances of 1.84 and 1.92 Å, respectively. Compound **2** interacted with Trp214, Glu248, and Phe250, as well as Trp206, and formed putative hydrogen bonds with Trp214, Glu248, and Phe250, with distances of 2.17, 2.45, and 2.35 Å, respectively.

Compound **5**, which exhibited the most favorable binding energy among the derivatives evaluated, showed interactions with Phe250, Asn208, Trp214, and Phe252. Putative hydrogen bonds were identified with Phe250 (1.87 Å), Asn208 (2.38 Å), and Trp214 (1.90 Å). These interactions, together with their docking score, suggest favorable stabilization of compound 5 within the evaluated binding site. In comparison, isocordoin exhibited a binding energy of −6.1 kcal/mol and shared several interacting residues with the evaluated derivatives, including Trp214, Phe247, Trp206, and Phe250.

Molecular docking against caspase-3 showed that all three derivatives exhibited favorable binding energies, with compound **5** displaying the most negative docking score (−6.4 kcal/mol), followed by compounds **2** (−6.1 kcal/mol) and **1** (−6.0 kcal/mol). Notably, compound **5**, which showed the highest proportion of caspase-positive cells in the flow cytometry assay, also exhibited the most favorable docking score among the derivatives evaluated. The docking results suggest that caspase-3 may represent a potential molecular target associated with the caspase activation observed experimentally. Previous studies have employed molecular docking against caspase-3 as an approach to explore the molecular interactions of natural products and relate them to cytotoxic and apoptotic activities [41].

From a structure interaction perspective, the predicted binding modes suggest that both the phenolic moieties and the hydrophobic carveol-derived scaffold contribute to ligand stabilization within the caspase-3 binding site. The phenolic hydroxyl groups of compounds **1**, **2**, and **5** participate in putative hydrogen-bond interactions with residues located in the binding region, including Phe250, Arg207, Glu248, Asn208, and Trp214. In parallel, the aromatic and hydrophobic portions of the molecules establish π-alkyl and hydrophobic contacts with aromatic residues such as Trp206, Trp214, Phe250, and Phe252. These interactions may contribute to the predicted stabilization of the compounds within the binding site. Notably, compound **5**, which contains two phenolic hydroxyl groups, showed the most favorable docking score (−6.4 kcal/mol), although these docking results alone do not establish a direct relationship between a specific structural feature and the experimentally observed caspase activation.

Nevertheless, docking scores should be interpreted as complementary structural evidence rather than as proof of enzymatic inhibition or activation. Therefore, the relationship between the predicted interactions and the experimentally observed caspase activation should be further validated using specific biochemical assays.

### 2.5. Physicochemical and Pharmacokinetic Properties

**Figure 5:**
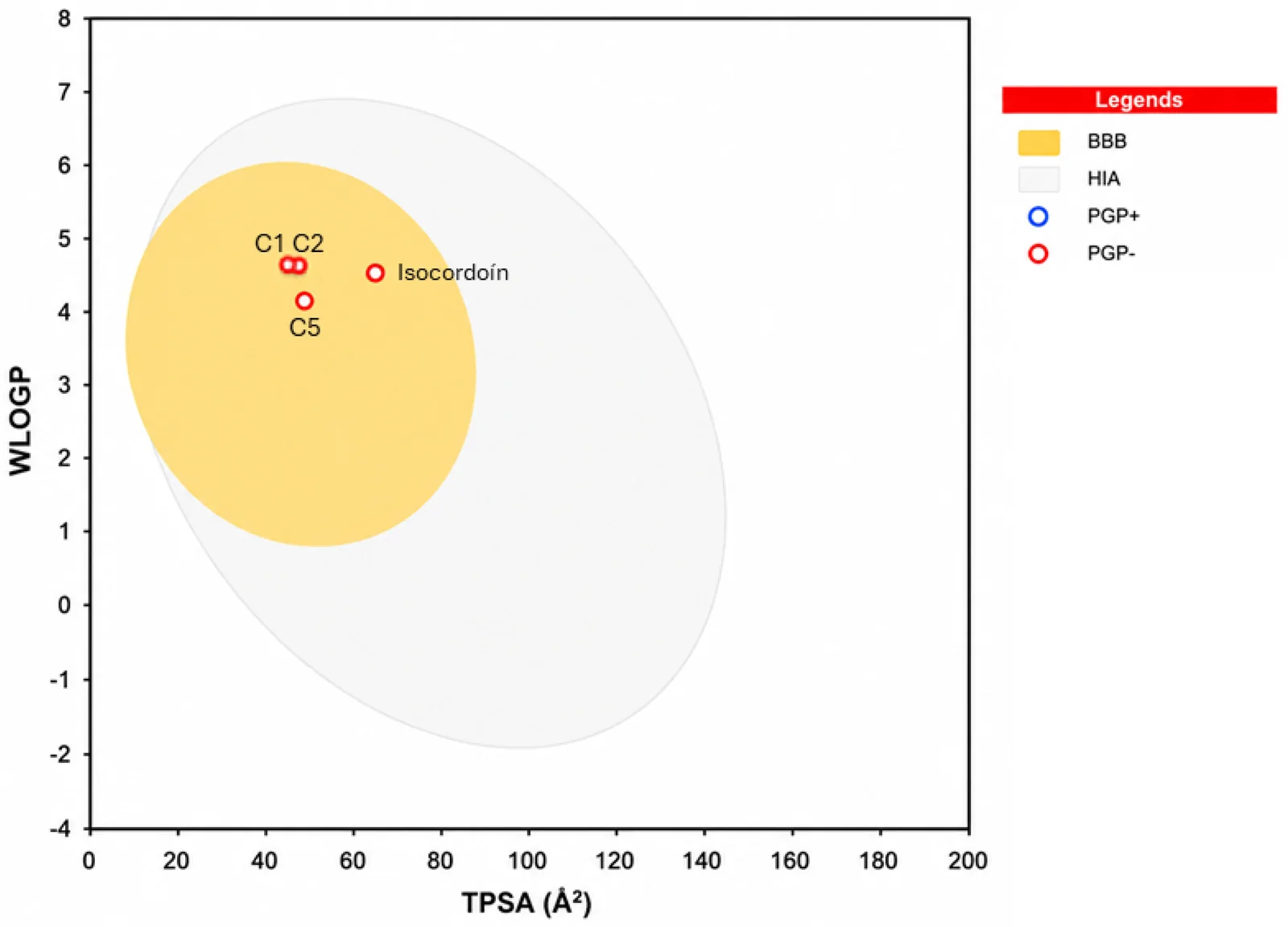
Prediction of Gastrointestinal Absorption, Blood–Brain Barrier Permeability, and P-gp Substrate Activity of Compounds **1**, **2**, **5**, and Isocordoin Using the SwissADME BOILED-Egg Model.

The SwissADME analysis revealed moderate differences in the physicochemical properties of compounds **1**, **2**, and **5** compared with isocordoin (Table 4). The derivatives exhibited WLOGP values lower than isocordoin, although their WLOGP values remained within a relatively lipophilic range. Overall, the three derivatives showed relatively similar physicochemical profiles, despite differences in the number of hydrogen-bond acceptors and donors and in structural flexibility. Regarding the predicted pharmacokinetic profile (Figure 5), all compounds exhibited high gastrointestinal absorption, potential blood–brain barrier (BBB) permeability, and no predicted P-glycoprotein (P-gp) substrate activity.

The observed differences in physicochemical properties may contribute to the differential biological behavior of the compounds. Compounds **1**, **2**, and **5** exhibited WLOGP values ranging from 3.67 to 4.03 and lower TPSA values than isocordoin, suggesting relatively greater lipophilicity and lower polarity. These features may favor interactions with hydrophobic environments and permeation across biological membranes, although increased lipophilicity may also limit aqueous solubility. Accordingly, the lower predicted solubility of isocordoin (LogS = −5.25) compared with the carveoylphenols may be associated with its higher WLOGP and TPSA values; however, these predictions do not establish a causal relationship with cytotoxic activity. The influence of lipophilicity, polarity, and solubility on the absorption and distribution of bioactive compounds is an important consideration in the early evaluation of candidates with pharmacological potential [42].

### 2.6. Drug-Likeness and Medicinal Chemistry Alerts

The *drug-likeness* analysis showed that compounds **1**, **2**, and **5** met the main medicinal chemistry criteria evaluated, whereas isocordoin exhibited a single alert under the Muegge criterion associated with its higher lipophilicity (Table 5). None of the compounds showed PAINS alerts, although both the derivatives and isocordoin displayed structural alerts in the Brenk filter. All compounds exhibited a Bioavailability Score of 0.55. Overall, these results indicate that compounds **1**, **2**, and **5** possess profiles compatible with general drug-likeness criteria, supporting their further evaluation using computational approaches.

Prediction using the SwissADME BOILED-Egg model showed that compounds **1**, **2**, and **5**, as well as isocordoin, were located within the yellow region associated with blood–brain barrier (BBB) permeability (Figure 5). Their position within the model’s defined area was also consistent with high gastrointestinal absorption. All four compounds were represented as red dots, indicating a predicted lack of P-glycoprotein (P-gp) substrate activity, in agreement with the SwissADME results (Table 5). Compounds **1, 2**, and **5** showed relatively similar distributions within the TPSA-WLOGP space, whereas isocordoin exhibited higher values for both descriptors while remaining within the region compatible with BBB permeability.

Overall, these results indicate that the three derivatives exhibit comparable physicochemical properties and predicted pharmacokinetic profiles, including high gastrointestinal absorption, potential BBB permeability, and no predicted P-gp substrate activity. Despite their differences in cytotoxicity, these derivatives displayed relatively similar physicochemical characteristics, suggesting that global parameters such as lipophilicity, polarity, and molecular weight alone do not fully account for the observed differences in activity. In this regard, specific structural features may play a key role in determining their interactions with cellular targets [43,44].

Therefore, the drug-likeness results provided initial support for the selection of these derivatives, while molecular docking against caspase-3 enabled further exploration of potential differences in their molecular interactions.

## 3. Materials and Methods

### 3.1. General

Carveol and solvents were purchased from Sigma-Aldrich (St. Louis, MO, USA) and used without further purification. Phenols and other reagents were obtained from AK Scientific (Union City, CA, USA) and used as received. NMR spectra were measured on a Bruker Avance 400 Digital NMR spectrometer in CDCl_3_ with TMS as the internal standard. Column chromatography (CC) was performed with silica gel 60 from Merck (Darmstadt, Germany), and thin-layer chromatography (TLC) was carried out on precoated silica plates F_254_ from Merck (Darmstadt, Germany).

### 3.2. General Procedure for the Synthesis of Carveol Derivatives (***1***–***5***, ***7***, ***9***, ***11***, ***12***)

The synthesis of carveol derivatives **1**–**5**, **7**, **9**, **11**, and **12** was carried out following a modified version of a previously reported protocol [19,31]. Reactions were performed using a CEM Discover 2.0 microwave synthesizer (CEM Corporation, Matthews, NC, USA). First, the catalyst was prepared by impregnating silica gel (SiO_2_) with a saturated solution of silver nitrate (AgNO_3_) in ethanol/water (1:1 *v*/*v*) and drying it prior to use, following previously reported methodology [31]. For each reaction, a 10 mL microwave-safe glass vial was loaded with carveol and the corresponding phenolic compound at a 2:1 molar ratio. The catalyst loading was adjusted to maintain the catalyst-to-alcohol ratio previously reported [19,31]. Prior to microwave irradiation, the reaction mixture was thoroughly homogenized by stirring, sealed with a Teflon cap, and placed in the reactor. Synthesis was conducted under optimized “Fixed Power” conditions (25 W, 55 °C) under low, constant stirring for 40 min in a single irradiation step without intermediate cooling, significantly reducing reaction times compared to classical procedures [19,31]. Upon completion, the vial was rapidly cooled using the equipment’s built-in compressed-air system. Reaction progress was monitored by thin-layer chromatography (TLC), accompanied by a visible color change in the mixture. To isolate the product and remove the solid catalyst, liquid–liquid extraction was performed using dichloromethane and a saturated sodium chloride solution in a separating funnel. The organic layer was dried over anhydrous MgSO_4_ and concentrated under reduced pressure via rotary evaporation. Finally, the crude product was purified by column chromatography on silica gel using a hexane/ethyl acetate elution gradient to yield pure compounds. Compound **12** represents a novel chemical entity reported here for the first time. All spectroscopic data for previously reported compounds (**1**–**5**, **7**, **9**, **11**) were in full agreement with the literature [19,32].

1-methyl-2-{[(5′*S*)-5′-isopropenyl-2′-methylcyclohex-2′-en-1-yl]oxy}-4-(isopropyl)benzene (**12**): Pale yellow viscous oil. Yield: 26.6%. ^1^HNMR (400.1 MHz, CDCl_3_): 7.07 (d, *J* = 7.6 Hz, 1H, H-5); 6.79 (s, 1H, H-4); 6.75 (d, *J* = 7.6 Hz, 1H, H-2); 5.75 (d, *J* = 5.2 Hz, 1H, H-3′); 4.72 (d, *J* = 4.1 Hz, 2H, H-8′); 4.60 (s, 1H, H-1′); 2.90–2.83 (m, 1H, H-7); 2.49 (t, *J* = 12.3 Hz, 1H, H-5′); 2.26–2.20 (m, 1H, H-6′β); 2.20 (s, 3H, H-10); 2.14–2.10 (m, 1H, H-4′β); 1.94–1.91 (m, 1H, H-6′α); 1.85 (s, 3H, H-10′); 1.73 (s, 3H, H-9′); 1.64–1.51 (m, 1H, H-4′α); 1.25 (d, *J* = 6.9 Hz, 6H, H-8 and H-9). ^13^C NMR (100.6 MHz, CDCl_3_): 156.6 (C-1); 149.2 (C-7′); 147.8 (C-3); 132.4 (C-2′); 130.6 (C-5); 126.6 (C-3′); 125.5 (C-6); 118.2 (C-4); 112.0 (C-2); 109.1 (C-8′); 75.0 (C-1′); 35.9 (C-5′); 34.1 (C-7); 32.7 (C-6′); 31.1 (C-4′); 24.1 (C-8 and C-9); 21.1 (C-10′); 20.9 (C-9′); 16.2 (C-10). EI-MS, *m*/*z* (%): 284 (12), 277 (23), 275 (10), 261 (63), 251 (12), 245 (22), 229 (22), 221 (15), 205 (37), 204 (100), 193 (16), 175 (18).

### 3.3. General Procedure for the Acetylation of Carveoylphenols

Acetylation was attempted across all crude reaction mixtures resulting from the coupling of carveol with phenols **I**–**VIII**, but product isolation was achievable exclusively for derivatives **6**, **8**, and **10**. NMR spectra for known adducts **6** and **10** matched literature-reported values [34]. Briefly, adapting a procedure by Madrid et al. [45], DMAP (1.0 mg) and Ac_2_O were added to a solution of the corresponding phenol derivative (1.0 equiv) in dry CH_2_Cl_2_. The precursor-to-Ac_2_O molar ratio was set based on free hydroxyl groups: 1:2 for **5** (yielding **6**), 1:3 for **7** (yielding **8**), and 1:1 for **9** (yielding **10**). After stirring at room temperature for 2 h, a cold 10% KHSO_4_ solution was added, and the aqueous layer was discarded. The organic phase was washed to neutrality with saturated NaHCO_3_ and water, dried over anhydrous MgSO_4_, filtered, and evaporated under reduced pressure. Purification by silica gel column chromatography (hexane/ethyl acetate, 8:2) afforded pure acetylated derivatives **6**, **8**, and **10**. Spectroscopic NMR data for previously reported derivatives **6** and **10** matched literature values [34]. Compound **8** represents a novel chemical entity and is reported here for the first time.

2-[(*5′R*)-5′-isopropenyl-2′-methylcyclohex-2′-en-1-yl]benzene-1,3,5-triacetate (**8**): Yellow oil; yield: 92%. ^1^HNMR (400.1 MHz, CDCl_3_): 6.84–6.80 (m, 2H, H-3 and H-5); 5.56–5.53 (m, 1H, H-3′); 4.71 (d, *J* = 6.1, 2H, H-8′); 3.55–3.34 (m, 1H, H-1′); 2.47–2.44 (m, 1H, H-5′); 2.35–2.04 (m, 1H, H-4′); 2.28 (s, 6H, 2×CH_3_CO); 1.96 (s, 3H, CH_3_CO); 1.94–1.82 (m, 1H, H-6′); 1.79 (s, 3H, H-9′); 1.36 (s, 3H, H-10′). ^13^C NMR (100.6 MHz, CDCl_3_): 168.9 (CH_3_CO); 168.5 (CH_3_CO); 168.1 (CH_3_CO); 149.6 (C-7′); 149.2 (C-4); 148.6 (C-6); 147.5 (C-2);133.9 (C-2′); 126.6 (C-3′); 122.0 (C-1); 115.0 (C-3); 113.6 (C-5); 109.1 (C-8′); 41.5 (C-5′); 34.9 (C-6′); 31.9 (C-4′); 29.6 (C-1′); 21.9 (C-9′); 21.6 (C-10′); 21.3 (CH_3_CO); 21.1 (CH_3_CO); 20.8 (CH_3_CO). EI-MS, *m*/*z* (%): 386 (10), 369 (14), 268 (42), 339 (23), 301 (31), 289(59), 275 (31), 261 (100), 251 (18), 239 (38), 235 (33), 219 (66), 207 (18), 190 (18), 186 (28), 177 (45).

### 3.4. Cell Culture

Cells were plated at 5 × 10^3^ cells/well in 100 μL of complete DMEM/F-12 medium using 96-well microplates. After a 24-h attachment period at 37 °C under 5% CO_2_ and humidified conditions, the cultures were treated with serial dilutions of the synthesized compounds and incubated for a further 72 h. The human cell lines used in this study were CoN CCD841 (normal colon epithelial cells), PC-3 (prostate adenocarcinoma), MCF-7 (breast adenocarcinoma), and HT-29 (colorectal adenocarcinoma), obtained from ATCC (Rockville, MD, USA). The culture medium was supplemented with 10% FBS, 100 U/mL penicillin, 100 μg/mL streptomycin, and 1 mM L-glutamine. Compounds were initially dissolved in DMSO, maintaining a final solvent concentration of 0.1% (*v*/*v*) in the assay. The vehicle control consisted of cells exposed to 0.1% DMSO under otherwise identical conditions [46].

### 3.5. Cytotoxicity Assay

The cytotoxic effects of the synthesized derivatives were determined by the sulforhodamine B (SRB) colorimetric assay (Sigma-Aldrich, St. Louis, MO, USA), according to a previously reported procedure with minor adaptations [47]. Cells were plated in flat-bottom 96-well plates at a density of 5 × 10^3^ cells/well in 100 μL of complete medium and allowed to attach for 24 h at 37 °C under a humidified atmosphere containing 5% CO_2_. The cells were subsequently treated with increasing concentrations of the compounds and incubated for 72 h.

At the end of the exposure period, cellular proteins were precipitated by adding cold trichloroacetic acid (TCA) to achieve a final concentration of 20%, using a 50% TCA stock solution maintained at 4 °C. The plates were then washed with distilled water and stained with 50 μL/well of 0.1% SRB prepared in 1% acetic acid for 30 min. Excess dye was eliminated by successive washes with 1% acetic acid, after which the protein-bound SRB was dissolved in 100 μL of 10 mM unbuffered Tris base. Absorbance was subsequently measured at 540 nm using a BioTek EL808 microplate reader [48].

Cell viability was expressed as a percentage relative to the vehicle-treated control according to the following equation:

CV (%) = ((A sample))/((A control)) × 100

where A sample denotes the absorbance obtained for the cells treated with each compound and A control corresponds to the mean absorbance of cells exposed to 0.1% DMSO. Untreated cells were included as negative controls, while daunorubicin and 5-fluorouracil were used as reference cytotoxic agents. The synthesized derivatives were tested at concentrations ranging from 0 to 100 μM, with DMSO used as the vehicle. Data is presented as mean ± standard deviation (SD) from three independent experiments, each performed in triplicate. IC_50_ values were obtained by nonlinear regression analysis using *R* software v4.6.1.

The selectivity index (SI) was calculated as the ratio between the IC_50_ value obtained in the non-tumorigenic reference cell line and that determined in each cancer cell line. CoN cells, a non-tumorigenic human colon epithelial cell line, were used as the reference for calculating the SI values for PC-3, MCF-7, and HT-29 cells. According to the established criterion, an SI > 2 was considered indicative of preferential cytotoxicity toward cancer cells, whereas an SI < 2 was interpreted as low selectivity, suggesting comparable cytotoxic effects toward tumor and non-tumorigenic cells [46,47,48].

### 3.6. Quantification of Reactive Oxygen Species (ROS)

Intracellular ROS levels were evaluated by flow cytometry using 2′,7′-dichlorofluorescein diacetate (DCFH-DA), according to the procedure reported by Silva et al. [49]. Cells were seeded in 24-well plates at 3 × 10^4^ cells/well and subsequently exposed to each compound at 20 and 40 µM concentrations. After treatment, DCFH-DA was added and the cells were incubated for 60 min at 37 °C. Unlabeled cells were included as the negative control, while solvent-treated and DCFH-DA-labeled cells were used as the vehicle control. Fluorescence was recorded on the FL1 channel, and the percentage of ROS-positive cells was determined from histogram analysis performed with FlowJo software v7.6.2.

### 3.7. Assessment of Mitochondrial Membrane Permeability

Mitochondrial membrane integrity was examined by flow cytometry following a previously reported procedure with adaptations [49]. Cells were seeded at 3 × 10^4^ cells/well in a final volume of 500 μL of culture medium and maintained for 24 h prior to treatment. The cells were then exposed to each compound at 20 and 40 µM concentrations for 48 h. Following treatment, mitochondrial membrane status was evaluated using 5 μL/well of Rhodamine 123, followed by incubation for 1 h at 37 °C. The proportion of cells exhibiting intact mitochondrial membrane permeability was determined by flow cytometry using the FL1 channel, and population analysis was performed with FlowJo software v7.6.2.

### 3.8. Determination of Caspase Activation

Caspase activation was investigated using the pan-caspase fluorescent probe FITC-VAD-FMK (CaspACE™). Cells were plated at 3 × 10^4^ cells/well in 500 μL of culture medium and allowed to adhere for 24 h before treatment. Subsequently, the cells were incubated with each compound at 20 and 40 µM for 48 h. Caspase activity was detected by fluorescent labeling for 1 h at 37 °C under dark conditions. Unlabeled cells were included to assess autofluorescence, whereas labeled cells treated with the vehicle served as the control. The percentage of caspase-positive cells was determined by histogram analysis of the FL1 fluorescence signal using FlowJo software v7.6.2 [49].

### 3.9. Molecular Docking and In Silico Analysis

#### 3.9.1. Ligand Preparation

Three-dimensional structures of the compounds were generated using Avogadro version 1.2.0. The resulting molecular geometries were optimized, and their energies were minimized using the MMFF94 force field prior to the docking calculations.

#### 3.9.2. Molecular Docking

Molecular docking was performed to investigate the potential interactions of the synthesized carveoylphenols and isocordoin with the catalytic region of caspase-3. The three-dimensional crystallographic structure of caspase-3 (PDB ID: 1NME), containing 2-hydroxy-5-(2-mercaptoethylsulfamoyl)benzoic acid (NLBA) as the co-crystallized ligand, was retrieved from the Protein Data Bank (RCSB PDB) [50].

Prior to docking, the protein structure was prepared using Discovery Studio Visualizer version 17.2.0.16349 (2016). The co-crystallized ligand and water molecules associated with the crystallographic structure were removed. Docking simulations were subsequently performed using AutoDock 4.2 and the Lamarckian genetic algorithm [51], considering the protein as a rigid receptor and allowing full conformational flexibility of the ligands. Kollman charges were assigned to the protein, whereas Gasteiger charges were assigned to the ligands. Polar hydrogen atoms were also added to the molecular structures.

The docking search space was defined using a grid centered at 40.881 Å (X), 95.736 Å (Y), and 24.611 Å (Z). The grid box comprised 45 × 40 × 40 points along the X, Y, and Z axes, respectively [49]. For each ligand, 50 independent docking runs were performed, with a maximum of 25,000,000 energy evaluations per run. The RMSD tolerance for conformational clustering was set to < 0.5 Å. The resulting poses were ranked according to their predicted binding energy and conformational clustering. The lowest energy pose from the most favorable cluster was selected for subsequent analysis of ligand-protein interactions. The use of the Lamarckian genetic algorithm and the grid-based search implemented in AutoDock 4.2 follows the established AutoDock methodology.

Two-dimensional interaction diagrams and three-dimensional representations of the selected ligand pose within the caspase-3 binding site were generated using Discovery Studio Visualizer version 21.1.0.20298.

#### 3.9.3. Validation of the Docking Protocol by Redocking

The reliability of the docking protocol was assessed by redocking the co-crystallized ligand NLBA into the caspase-3 binding site. The resulting redocked conformation was compared with the experimentally determined crystallographic pose by calculating the root mean square deviation (RMSD) between the corresponding ligand coordinates.

The redocking procedure yielded an RMSD value of 1.7 Å, indicating good reproduction of the experimentally observed binding orientation of the co-crystallized ligand. This value was considered acceptable for validating the docking protocol prior to the analysis of the synthesized derivatives. The use of redocking as a validation strategy is consistent with established molecular docking protocols, in which the ability of a method to reproduce the crystallographic ligand pose is assessed by RMSD.

#### 3.9.4. Prediction of Physicochemical, Pharmacokinetic, and Drug-Likeness Properties

The physicochemical, lipophilic, pharmacokinetic, and drug-likeness properties of the synthesized compounds were predicted using SwissADME (http://www.swissadme.ch/index.php), accessed on 25 August 2026.

## 4. Conclusions

Carveoylphenols exhibited structure- and cell line-dependent cytotoxic activity, with compound **2** showing the highest potency. While flow cytometric analyses provided preliminary insights suggesting the potential involvement of oxidative stress, mitochondrial dysfunction, and caspase activation, further studies are required to establish a detailed mechanism of action and evaluate cancer-cell selectivity. Furthermore, in silico analyses revealed favorable physicochemical and drug-likeness profiles for compounds 1, 2, and 5. Overall, these findings position carveoylphenols as preliminary candidates for further structural optimization and in-depth mechanistic investigation.

## Figures and Tables

**Table 1:** In vitro cytotoxic activity (IC_50_, µM) of carveoylphenols (**1**–**12**), natural reference, and reference drugs against human cancer and non-tumorigenic cell lines.

Compounds	Cell Lines IC_50_ (µM)			
	CoN	PC-3	MCF-7	HT-29
**1**	49.9 ± 0.8	62.3 ± 18	52.4 ± 6.8	46.4 ± 7.8
**2**	32.1 ± 6.0	26.5 ± 6.2	30.9 ± 3.3	23.2 ± 5.0
**3**	95.2 ± 4.7	>100	>100	>100
**4**	>100	>100	>100	>100
**5**	69.9 ± 21.3	79.0 ± 19.8	66.9 ± 10.3	54.9 ± 18.8
**6**	37.5 ± 3.2	41.3 ± 2.4	47.2 ± 5.2	34.5 ± 1.7
**7**	50.4 ± 3.5	67.2 ± 15.3	70.6 ± 10.5	47.4 ± 13.5
**8**	57.5 ± 2.9	75.3 ± 14.9	>100	54.3 ± 20.9
**9**	>100	>100	>100	85.1 ± 17.9
**10**	>100	>100	>100	>100
**11**	44.1 ± 4.1	59.6 ± 7.6	58.5 ± 7.3	61.8 ± 9.9
**12**	>100	>100	>100	>100
**Isocordoin**	78.7 ± 0.1	15.2 ± 0.6	21.1 ± 0.2	27.2 ± 0.3
**Daunorubicin**	13.9 ± 0.3	0.41 ± 0.4	0.33 ± 0.2	15.1 ± 0.5
**5-Fu**	56.1 ± 0.5	16.4 ± 0.6	22.3 ± 0.2	2.9 ± 0.7

IC_50_ values are expressed in µM. CoN represents the non-tumorigenic colon cell line, whereas PC-3, MCF-7, and HT-29 correspond to prostate, breast, and colon cancer cell lines, respectively. Data are presented as mean ± standard deviation (SD). Values reported as >100 µM indicate concentrations above the maximum concentration tested.

**Table 2:** Selectivity indices (SI) of compounds **1**–**12**, natural reference, and commercial controls against evaluated human cancer cell lines.

Compounds	PC-3	MCF-7	HT-29
**1**	0.8	0.95	1.08
**2**	1.21	1.04	1.38
**3**	<0.95	<0.95	<0.95
**4**	ND	ND	ND
**5**	0.88	1.04	1.27
**6**	0.91	0.79	1.09
**7**	0.75	0.71	1.06
**8**	0.76	<0.58	1.06
**9**	ND	ND	>1.18
**10**	ND	ND	ND
**11**	0.74	0.75	0.71
**12**	ND	ND	ND
**Isocordoin**	5.18	3.73	2.89
**Daunorubicin**	33.9	42.12	0.92
**5-Fu**	3.42	2.52	19.34

The selectivity index (SI) was calculated as the ratio of the IC_50_ value obtained for the non-tumorigenic CoN CCD841 cell line to that obtained for each corresponding tumor cell line [SI = IC_50_(CoN)/IC_50_ (tumor cell line)]. SI values >1 indicate greater sensitivity of the tumor cell line relative to CCD841, whereas values <1 indicate greater relative sensitivity of the non-tumorigenic cell line. ND: not determined due to IC_50_ values >100 µM.

**Table 3:** Predicted binding energies and molecular interactions of compounds **1**, **2**, and **5** with caspase-3 (PDB: 1NME).

Compound	Docking Score (ΔG) (kcal/mol)	Amino Acids Involved
**1**	−6.0	Phe250 (1.84), Arg207 (1.92), Trp206, Arg207
**2**	−6.1	Trp214 (2.17), Glu248 (2.45), Phe250 (2.35), Trp206, Phe250
**5**	−6.4	Phe250 (1.87), Asn208 (2.38), Trp214 (1.90), Phe252
NLBA *	−5.5	Arg207, Tyr204, Asn208, Phe250, Ser209
**Isocordoin**	−6.1	Phe256, Trp214, Phe247, Trp 206, Phe250
**Daunorrubicin**	−7.1	Tyr204, Arg207, Ser205, Phe252
**5-FU**	−3.7	Asn208, Trp214, Arg207, Phe250

* NLBA: 2-hydroxy-5-(2-mercaptoethylsulfamoyl)benzoic acid. Values in parentheses correspond to the distances of the putative hydrogen bonds, expressed in Å.

**Table 4:** Physicochemical Properties of the Compounds Evaluated Using SwissADME.

Compound	MW	TPSA	HBA	HBD	RB	WLOGP	LogS
**1**	258.36	40.46	2	2	2	4.03	−4.36
**2**	288.38	38.69	3	1	4	3.94	−4.35
**5**	244.33	40.46	2	2	2	3.67	−4.05
**Isocordoin**	308.37	57.53	3	2	5	4.46	−5.25

MW, molecular weight; TPSA, topological polar surface area; HBA, hydrogen-bond acceptors; HBD, hydrogen-bond donors; RB, rotatable bonds; WLOGP, octanol/water partition coefficient calculated using the Wildman-Crippen method; LogS (ESOL), estimated aqueous solubility.

**Table 5:** Evaluation of Drug-Likeness and Medicinal Chemistry Alerts.

Compound	Lipinski	Ghose	Veber	Egan	Muegge	PAINS	Brenk	Bioavailability Score
**1**	Yes	Yes	Yes	Yes	Yes	0	Isolated alkene	0.55
**2**	Yes	Yes	Yes	Yes	Yes	0	Isolated alkene	0.55
**5**	Yes	Yes	Yes	Yes	Yes	0	Isolated alkene	0.55
**Isocordoin**	Yes	Yes	Yes	Yes	XLOGP3 > 5	0	Isolated alkene, Michael acceptor	0.55

## Data Availability

The original contributions presented in this study are included in the article and Supplementary Materials. All cell lines analyzed were obtained from the American Type Culture Collection (ATCC, Rockville, MD, USA). For any further inquiries, please contact the corresponding author.

## Supplementary Materials

The following supporting information can be downloaded at: https://www.mdpi.com/article/10.3390/ijms27198749/s1.
