# Supplementary material for: Microwave-Assisted Synthesis, Cytotoxic Profile, and Cellular Mechanism Analysis of Carveoylphenol Derivatives
Source: Int J Mol Sci. 2026 Sep 30;27(19):8749. doi: 10.3390/ijms27198749 (PMC13665034; doi:10.3390/ijms27198749)

SUPPORTING INFORMATION

Microwave-Assisted Synthesis, Cytotoxic Profile, and Cellular Mechanism Analysis of Carveoylphenol Derivatives

Evelyn Muñoz [1], Constanza Reyes [2], Joan Villena [3], Rut Vergara [3], Guillermo Bravo [4] and Alejandro Madrid [2,*]

^{1}H NMR spectrum of compound **8**

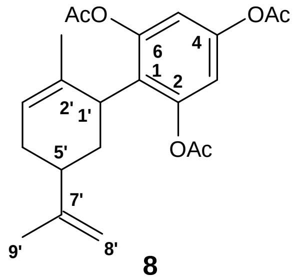

8

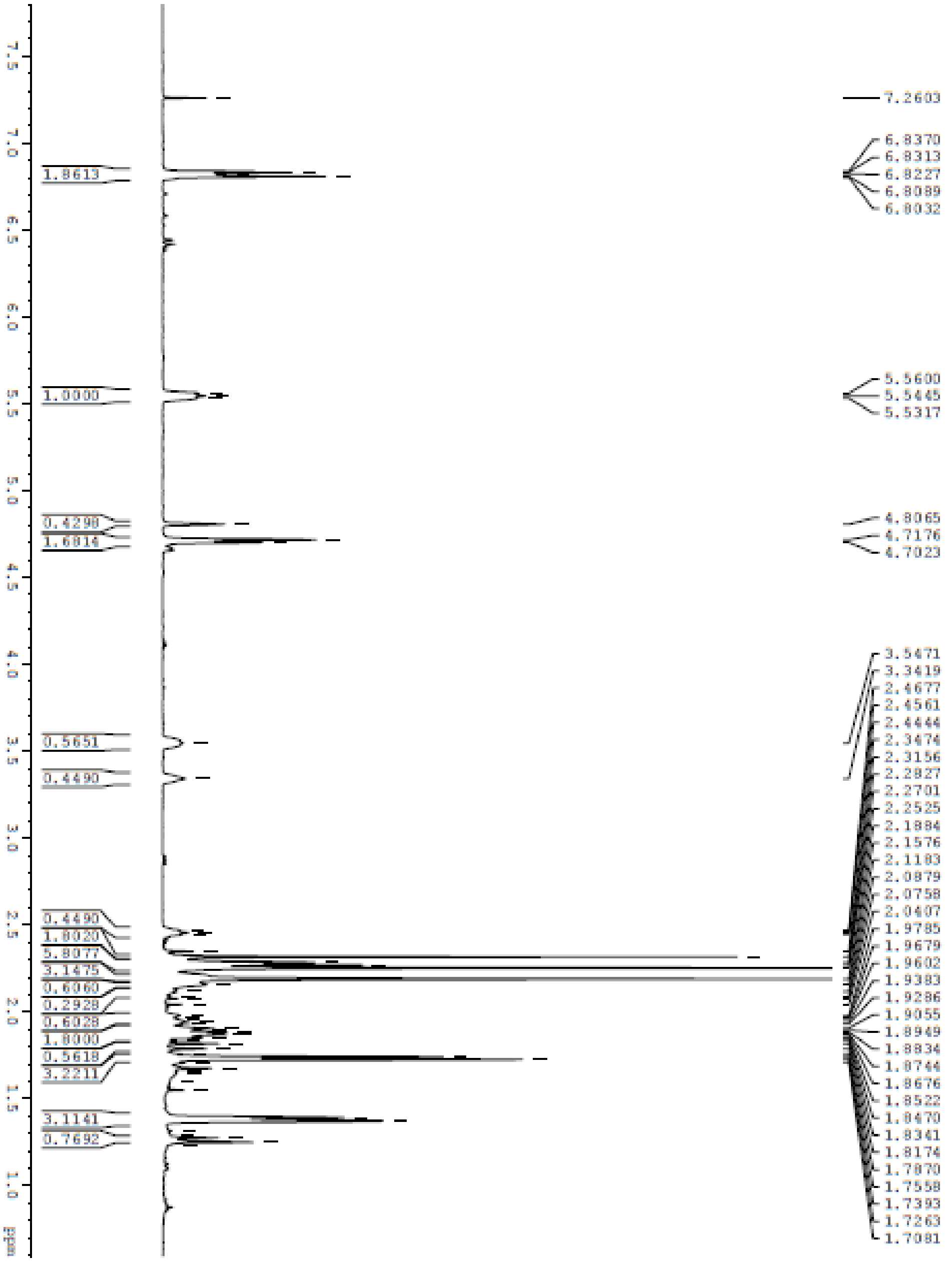

^{13}C NMR spectrum of compound **8**

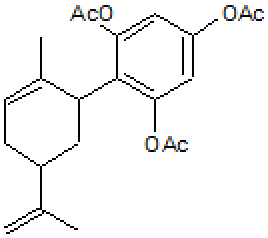

8

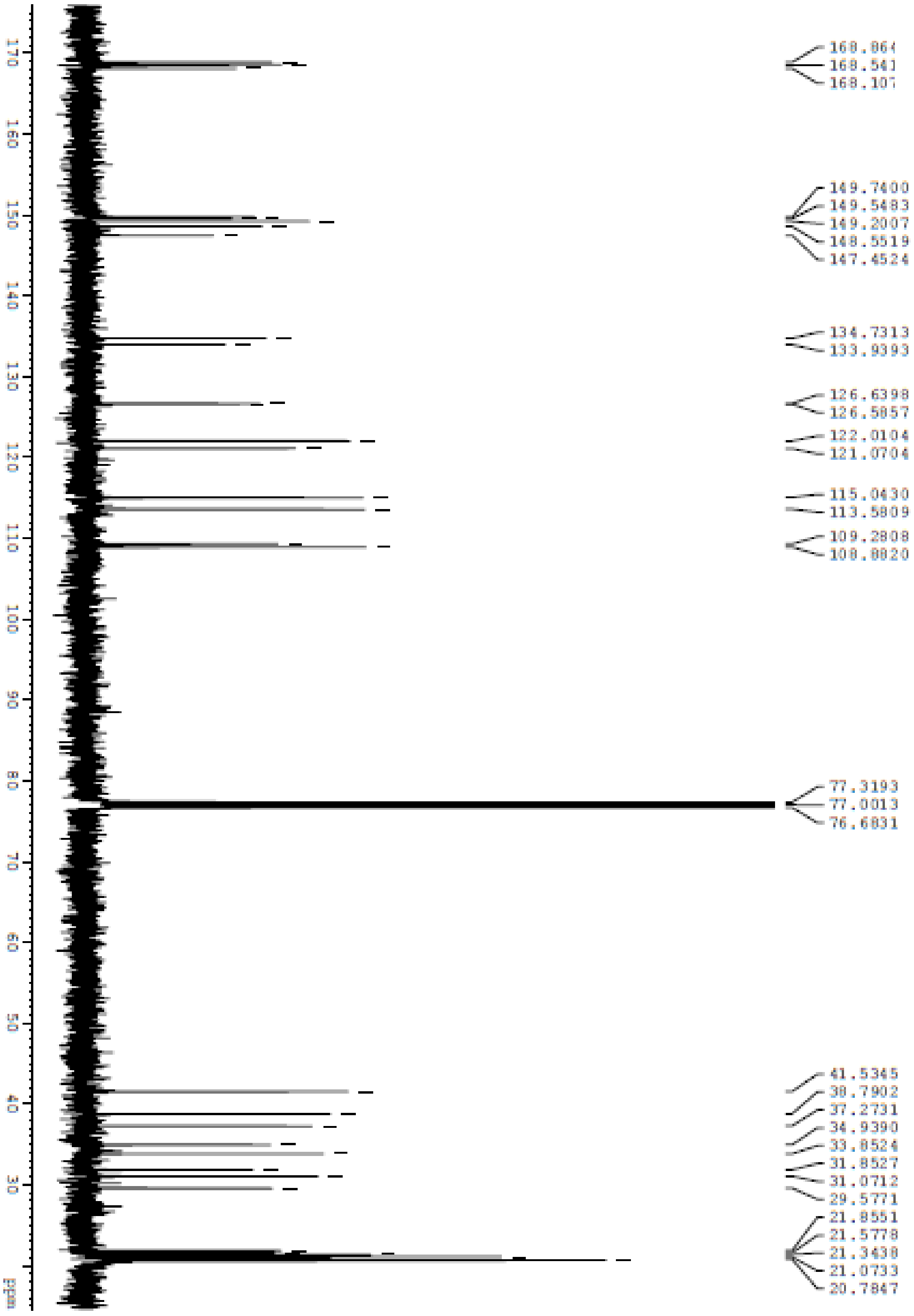

Mass spectra of compound **8**

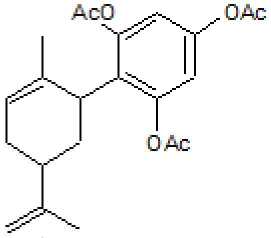

8

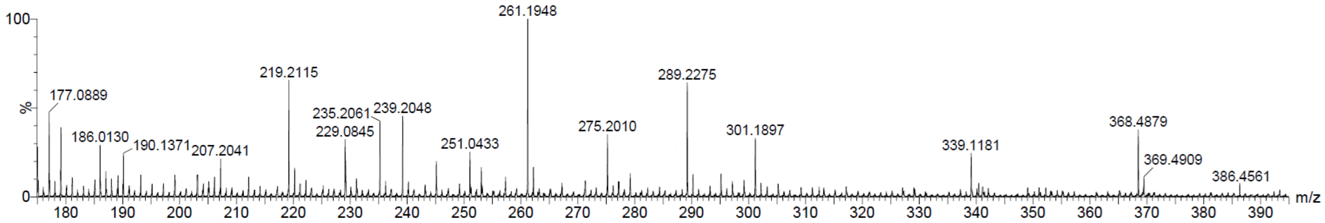

^{1}H NMR spectrum of compound **12**

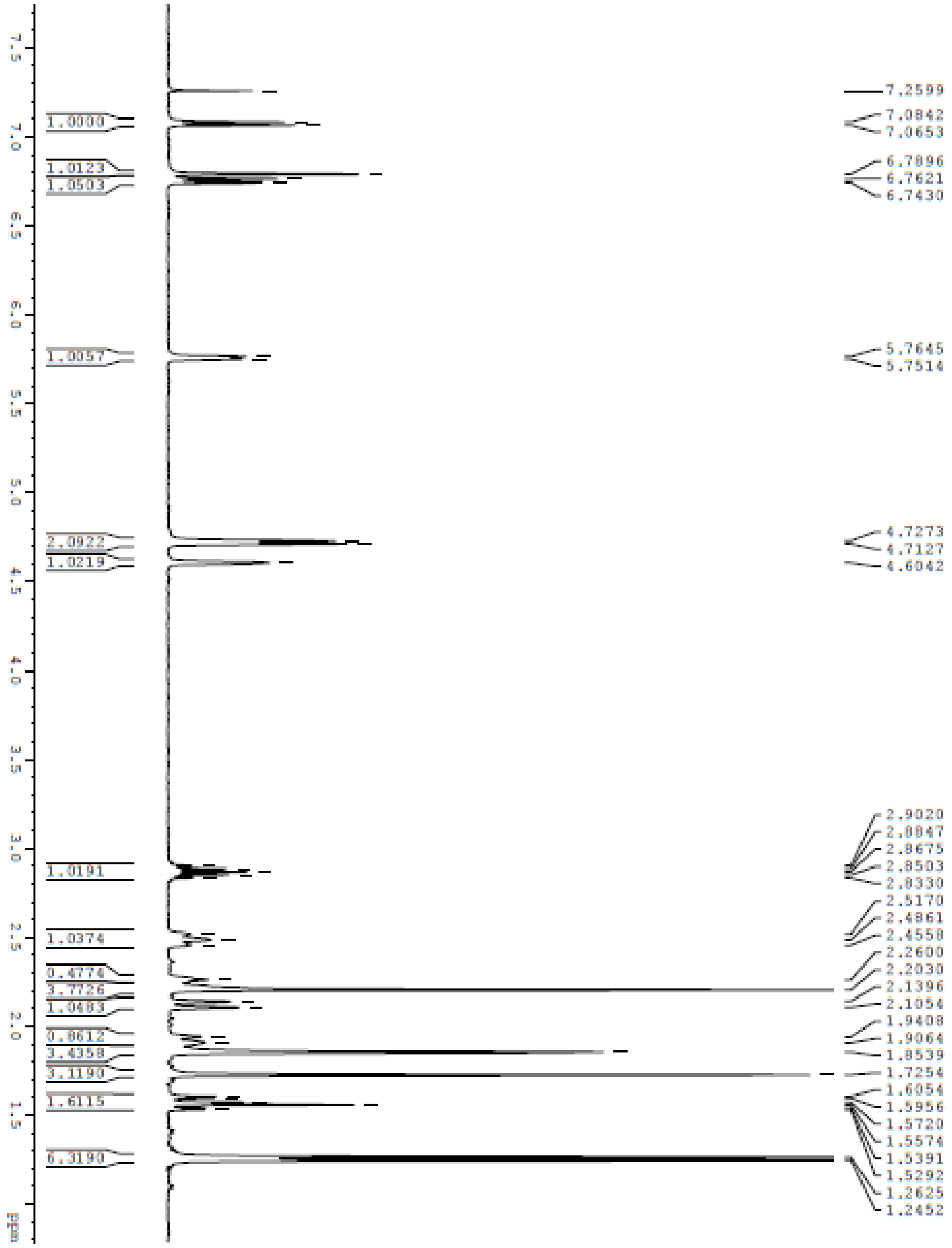

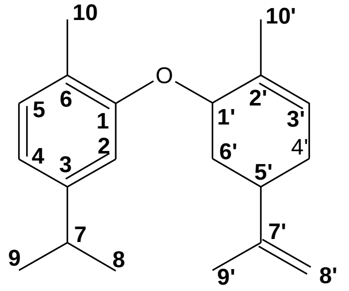

12

^{13}C NMR spectrum of compound **12**

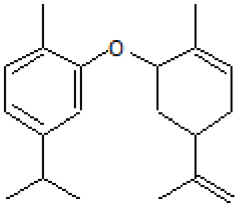

12

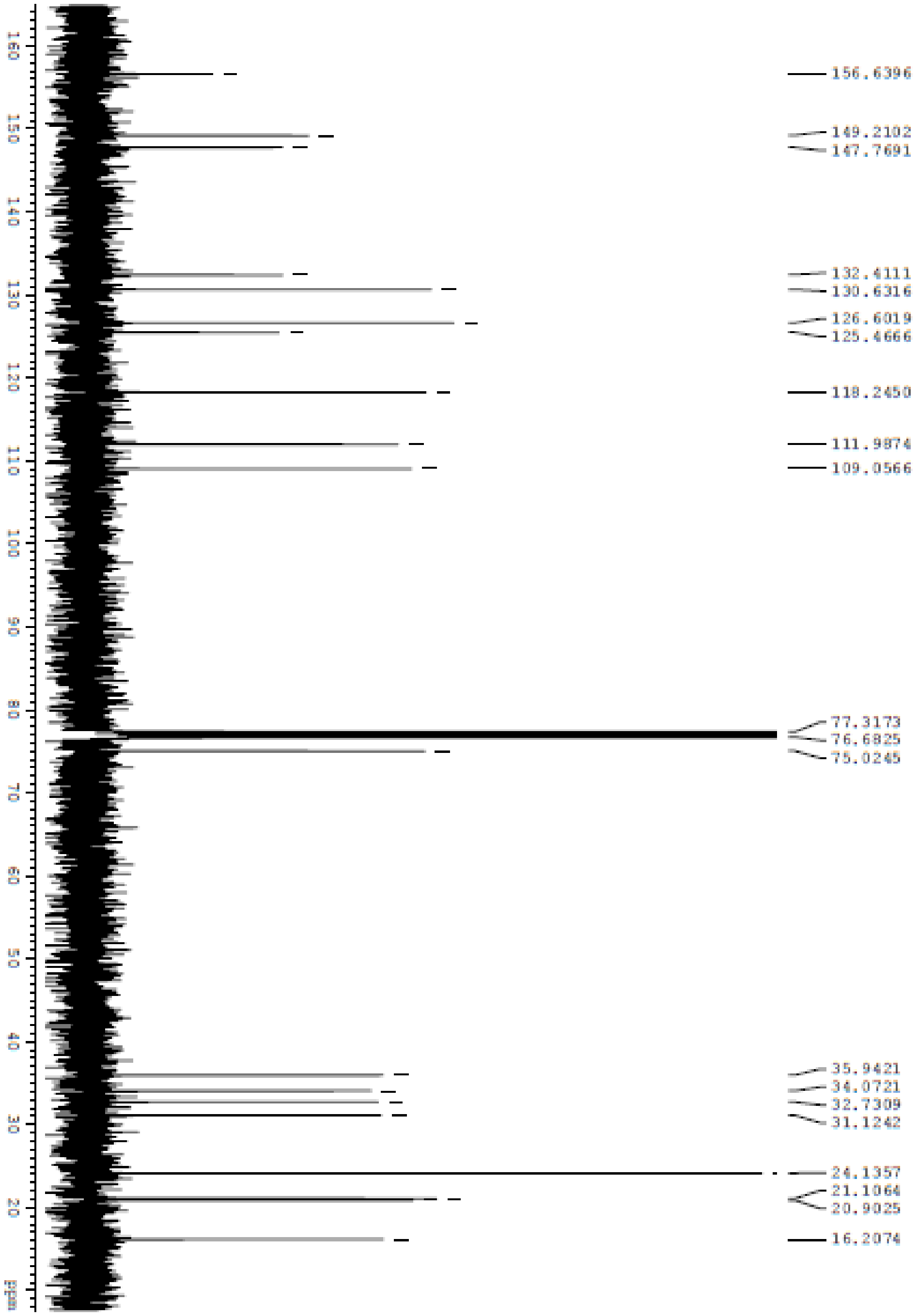

Mass spectra of compound **12**

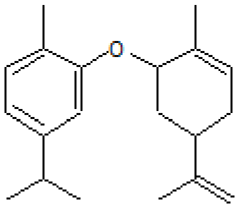

12

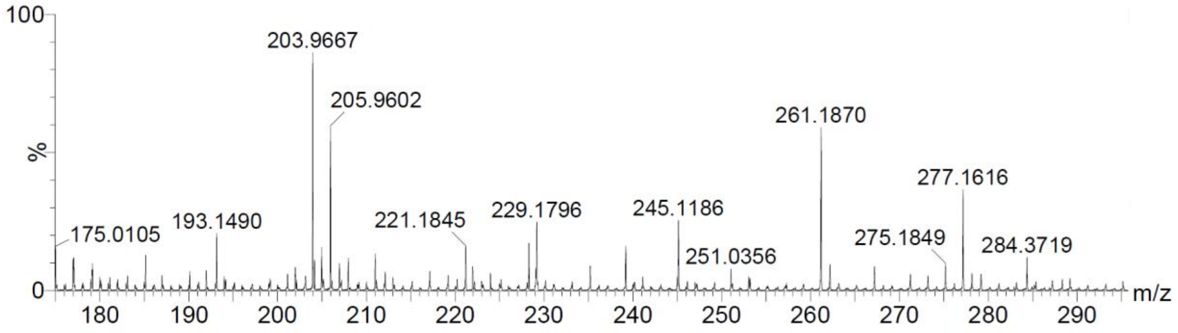

Supplement: Supplementary file 1 [file ijms-27-08749-s001.zip › ijms-4578517-supplementary.pdf]
